# Use of Cardiovascular Disease Secondary Prevention Medications in Four Middle East Countries in a Community Setting

**DOI:** 10.5334/gh.1349

**Published:** 2024-08-26

**Authors:** Afzalhussein Yusufali, Marwan Zidan, Rasha Khatib, Roya Kelishadi, khalid Alhabib, Mariam Alnoman Alshamsi, Ahmad Farid Rais, Afra Khalid Bintouq, Ahmad Bahonar, Noushin Mohammadifard, Mostafa Al Shamiri, Sumathy Rangarajan, Hamda Khansaheb, Salim Yusuf

**Affiliations:** 1 Health Improvement Project Zanzibar, Zanzibar, United Republic of Tanzania; 2 Dubai Health, Dubai, United Arab Emirates; 3 Academic Research and Strategic Partnerships, Advocate Aurora Research Institute (AARI), Milwaukee, USA; 4 Isfahan Cardiovascular Research Center, Cardiovascular Research Institute, Isfahan University of Medical Sciences, Isfahan, Iran; 5 College of Medicine, Department of Cardiac Sciences, King Fahad Cardiac Center, King Saud University, Riyadh, Saudi Arabia; 6 Population Health Research Institute, McMaster University, Hamilton Health Sciences, Hamilton, ON, Canada

**Keywords:** Middle East, secondary prevention, cardiovascular disease, evidence based medications

## Abstract

**Background:**

Evidence-based International clinical practice guidelines, universally recommend secondary prevention medications for those with previous cardiovascular disease (CVD). There is limited data on the community use of these medications in the Middle East (ME).

**Objectives:**

This study assesses the use and predictors of evidence based secondary prevention medications in individuals with a history of CVD [coronary heart disease (CHD) or stroke].

**Methods:**

Between 2005 and 2015, we enrolled 11,228 individuals aged between 35–70 years from 52 urban and 35 rural communities from four ME countries, United Arab Emirates (n = 1499), Kingdom of Saudi Arabia (n = 2046), Occupied Palestinian Territory (n = 1668) and Islamic Republic of Iran (n = 6013). With standardized questionnaires, we report estimates of medication use in those with CVD at national level and the independent predictors of their utilization through a multivariable analysis model. Results: Of the total ME cohort, 614 (5.5%) had CVD, of which 115 (1.0%) had stroke, 523 (4.7%) had CHD and 24 (0.2%) had both. The mean age of those with CVD was 56.6 ± 8.8 years and 269 (43.8%) were female. Overall, only 23.5% of those with CVD reported using three or more proven secondary prevention medications, and a substantial proportion (stroke 27.8%, CHD 25.8%) did not take any of these medications. In a fully adjusted analysis, increasing age, female gender, higher education, higher wealth in individual household, residence in a higher income country as well as being obese, hypertensive or diabetic were independent predictors of medication use.

**Conclusion:**

The use of secondary prevention medication is low in ME and has not reached the modest recommended WHO target of 50% use of 3 or more medications. Independent factors of higher use were, better socioeconomic status (household wealth, country wealth and education) and better contact and accessibility to health care (increasing age, female gender, obesity, diabetes and hypertension).

## Introduction

Cardiovascular disease (CVD) is the leading cause of death worldwide (1). In 2019, prevalent CVD reached around 523 million (2), and it takes around 17.9 million lives each year (3). In the Middle East (ME), it is also the most common cause of death. Non-communicable diseases account for 82% of all deaths in Iran, and almost half are due to CVD, while in UAE and Saudi Arabia, 40% and 37% respectively of all deaths are due to CVD (4).

Half of the CVD events occur in those with previous CVD (5). After coronary heart disease (CHD) or stroke, the recurrence rate of other CVD events or death is very high. In one recent Finnish registry, the five years post-index cardiovascular event, death, or recurrent cardiovascular event was 41.5% (6).

Evidence-based CVD secondary prevention medications (such as anti-platelets, statins, beta-blockers, angiotensin-converting enzyme inhibitors (ACE-I) or angiotensin-receptor blockers (ARBs)) in CHD and antiplatelets, statins, and any of the antihypertensive drugs (like beta-blockers, ACE-I or ARBs, calcium channel blockers or diuretics) in stroke (7,8,9,10,11,12) is expected to reduce the recurrence of the event or death by 60 to 80% (13,14). Based on this evidence, international clinical practice guidelines universally recommend these secondary prevention medications for those with previous CHD or stroke (15,16). Despite these recommendations, their implementation globally, especially among low- and middle-income countries, and in rural areas remains less than ideal (17,18).

Data on rates of secondary prevention medication use and their predictors in the Middle East are sparse and are mainly evaluated using hospital or clinic registries (19,20,21,22,23). Four Middle Eastern countries, the United Arab Emirates (UAE) the Kingdom of Saudi Arabia (KSA), the Occupied Palestinian Territory (OPT), and the Islamic Republic of Iran (Iran) are part of the Prospective Urban Rural Epidemiology (PURE) study (24). PURE is a cohort study of communities living in urban and rural locations. This report uses PURE-Middle East (PURE-ME) baseline data to examine the rate of the utilization of evidence-based secondary prevention medications among patients with CVD, at the country, urban and rural community levels. It also examines the predictors of their use in all four countries combined.

### Study Design and Participants

The Prospective Urban Rural Epidemiology (PURE) study is a large-scale epidemiology cohort study that has recruited 225,000 participants between the ages of 35 to 70 years, residing in over 600 communities in 27 low income countries (LIC), middle income countries (MIC), and high-income countries (HIC) around the world. The overall aim of PURE was to examine the relationship between societal influences on lifestyle behaviours, cardiovascular risk factors, and incidence and mortality of chronic diseases. The design and methodology of the main PURE study have previously been described (24). In the Middle East, we enrolled 11,228 individuals from 52 urban and 35 rural communities, in the UAE (from 27/12/2005 till 11/05/2009), KSA (01/02/2012 till 01/10/2015), Iran (01/07/2006-01/10/2008), and OPT (09/03/2012 till 15/03/2013) and they continue to be followed up. This report uses data collected at baseline only.

### Ethics Statement

Ethical approval was obtained in UAE from Dubai Scientific Research Ethics Committee and extension provided on 15/02/2023 Ref: DSREC-01/2023-31, in KSA from King Saud University, Institutional Review Board Ref 24/0961/IRB extended on 04/01/2024, in Iran from Isfahan Cardiovascular Research Institute Ref:224/2006 on 09/03/2006 and in OPT from Birzeit University -The Committee on Research Ethics no.120514 on 04/04/2015. All eligible individuals provided written informed consent before recruitment.

## Procedures

This analysis was limited to the sub-cohort of PURE participants in the Middle East. Data was collected using standardized questionnaires that collected self-reported information on age, gender, education, smoking status, hypertension, diabetes, obesity, history of cardiovascular and other diseases, and medication use. Medication use was self-reported by collecting the names of all medications the participant was currently taking (Appendix I).

The outcomes in this analysis included the baseline rates of CVD and the baseline name and quality of evidence-based CVD medications that the participant reported taking. CVD was defined as self-reporting, at baseline, a history of stroke or history of coronary heart disease (CHD). CHD was defined as myocardial infarction, coronary artery bypass graft surgery, or percutaneous coronary angioplasty or angina. In addition to rates of total CVD, the rates of CHD and stroke are also reported separately. Baseline medications were grouped by class and included statins, anti-platelet medications, beta-blockers, angiotensin-converting enzyme inhibitors (ACE-I) or angiotensin-receptor blockers (ARBs), calcium channel blockers, and diuretics. We reported the use of each of these medications and the use of any blood pressure-lowering medications which included using at least one of the following medications: beta-blockers, ACE-I or ARBs, calcium channel blockers, or diuretics. We also reported the number of medications used (0,1,2, and 3 or more). We further categorize medication use in two ways, as ‘one or more medications used (>=1)’ versus ’no medication is used’, and as ’two or more medications used (>=2)’ versus ’none or one medication is used‘. A patient taking two medications of the same class was considered as taking one class of medication.

The predictors examined in this analysis included the CVD patients’ demographic characteristics: age, gender, education (none/primary or unknown, secondary or higher education, and college/trade/university education), and urban versus rural residence. We used data collected on household possessions (electricity supply and ownership of an auto- mobile, other four-wheel vehicle, computer, television, motorbike, livestock, refrigerator, washing machine, stereo, bicycle, kitchen mixer, telephone, land or real estate, and kitchen window) to generate an asset-based wealth index. It places households within each country-specific sample on a continuous scale of relative wealth from poorest to richest. Tertiles of the wealth index were used to categorize individuals into low, middle, and wealthy within each country (24). Patients’ clinical characteristics included hypertension diagnosis, diabetes diagnosis, body mass index (BMI; <25, 25 <=BMI <30 and >=30), and smoking status (never, former, and current). Countries were categorised into High income countries-HIC (KSI and UAE) and middle-income countries-MIC (OPT and Iran) according to the World Bank.

### Statistical Analysis

The rates of CHD, stroke, and total CVD are reported among all PURE-ME participants, while the use of secondary prevention medication is reported among the subgroup of patients who report a history of CHD or Stroke at baseline. Categorical variables, such as gender and use of each class of medication were presented as frequencies (%) with 95% confidence intervals.

Five binary logistic regression models were created to test the independent significant predictors of using each class of medication or at least one antihypertensive medication. An additional two binary logistic regression models were created to test independent significant predictors of using at least one class versus no medications and using more than one class versus one class or none. Results from the models are presented as odds ratio (OR) and 95% confidence intervals (CI).

Statistical significance is set at p-value <0.05. All the statistical analysis was performed using SPSS 24 (IBM SPSS Statistics for Windows, Version 24.0. Armonk, NY: IBM Corp).

## Results

Of the 11,226 participants included in the PURE-ME sub-cohort, 614 (5.5%) reported a history of CVD, of which 115 (1.0%) reported a history of stroke, 523 (4.7%) reported a history of CHD, while 24 (0.2%) had both. The median number of years since these events occurred was 4 years (IQR 2.0–8.3) for CHD and 4 years (IQR 2.0–8.0) for stroke.

OPT had the highest rates of total CVD at 6.8% (stroke 1.6% and CHD 6.0%), followed by Iran at 6.0% (stroke 1.0% and CHD 5.1%), the UAE at 4.8% (stroke 0.7% and CHD 4.2%), while KSA had the lowest rates at 3.4% (stroke 1.0% and CHD 2.5%) (**Supplementary Table 1**).

Of the total CVD (614), the mean age was 56.6 ± 8.8 years and 269 (43.8%) were female. Only 61(9.9%) were educated at trade or college/university level, and 416 (67.8%) were none or primary school educated, or their education status was unknown. The proportion of overweight and obese patients were 242 (40.7%) and 229 (38.6%) respectively, while current smokers were 126 (20.5%) and never smokers were 406 (66.1%). History of hypertension was in 354 (60.7%) and 280 (45.6%) were diabetic (Table 1).

**Table 1:** Characteristics of patients with coronary heart disease (523), stroke (115) and total CVD (n = 614).


FACTOR	CHD (523)	STROKE (115)	TOTAL CVD (614)				

	COUNT %	COUNT %	COUNTRY				

			UAE (72)	KSA (69)	OPT (114)	IRAN (359)	TOTAL (614)

**Age**	56.9 ± 8.4	55.7 ± 9.8	59.4 ± 8.8	55.2 ± 10.4	58.9 ±8.9	55.97 ± 8.2	56.6 ± 8.8

**Sex**							

Female	223 (42.6)	56 (48.7)	39 (54.2)	21 (30.4)	32 (28.1)	177 (49.3)	269 (43.8)

**Smoke History**							

Former Smoker	74 (14.1)	12 (10.4)	11 (15.3)	11 (15.9)	25 (21.9)	35 (9.7)	82 (13.4)

Current Smoke	112 (21.4)	21 (18.3)	8 (11.1)	9 (13)	32 (28.1)	77 (21.4)	126 (20.5)

Never Smoker	337 (64.4)	82 (71.3)	53 (73.6)	49 (71)	57 (50)	247 (68.8)	406 (66.1)

**Location**							

Urban	281 (53.7)	52 (45.2)	45 (62.5)	47 (68.1)	54 (47.4)	175 (48.7)	321 (52.3)

**Household Wealth Index Tertiles**							

Low	69 (13.2)	23 (20)	0 (0)	9 (13)	27 (23.7)	53 (14.8)	89 (14.5)

Middle	339 (64.8)	76 (66.1)	27 (37.5)	59 (85.5)	50 (43.9)	261 (72.7)	397 (64.7)

High	115 (22)	16 (13.9)	45 (62.5)	1 (1.4)	37 (32.5)	45 (12.5)	128 (20.8)

**Education**							

None, Primary, or Unknown	351 (67.1)	77 (67)	66 (91.7)	35 (50.7)	44 (38.6)	271 (75.5)	416 (67.8)

Secondary/High/Higher secondary	119 (22.8)	24 (20.9)	1 (1.4)	14 (20.3)	53 (46.5)	69 (19.2)	137 (22.3)

Trade or College/University	53 (10.1)	14 (12.2)	5 (6.9)	20 (29)	17 (14.9)	19 (5.3)	61 (9.9)

**BMI groups**							

0 < BMI < 25	106 (20.9)	21 (19.1)	15 (23.1)	11 (15.9)	11 (10.7)	86 (24.1)	123 (20.7)

25 <= BMI < 30	204 (40.2)	45 (40.9)	18 (27.7)	22 (31.9)	35 (34)	167 (46.8)	242 (40.7)

BMI >= 30	197 (38.9)	44 (40)	32 (49.2)	36 (52.2)	57 (55.3)	104 (29.1)	229 (38.6)

**Hypertension**	305 (61.1)	69 (64.5)	36 (73.5)	50 (72.5)	78 (73.6)	190 (52.9)	354 (60.7)

**Diabetes**	237 (45.3)	62 (53.9)	47 (65.3)	43 (62.3)	70 (61.4)	120 (33.4)	280 (45.6)

**Country Economic level**							

High-income country	114 (21.8)	30 (26.1)	72 (100)	69 (100)	–	–	141 (23)

Lower-income country	409 (78.2)	85 (73.9)	–	–	114 (100)	359 (100)	473 (77)


Categorical variables were presented as count (%), while age was presented as mean ±SD.

### Medication Use

Medication use is presented in Table 2. Blood pressure lowering medications and antiplatelet medications were the most used medications (62.7% and 47.6%, respectively), while 37.1% reported using statins. Medication use was greater among participants with CHD compared to participants with stroke across all medication classes. Antiplatelet medications were used among 49.1% of CHD participants and among 38.3% of stroke patients. Statins were used among 37.7% of CHD and among 33.9% of stroke patients. Beta-blockers were used among 41.3% of participants with CHD and 26.1% of participants with stroke. ACE inhibitors or ARBs were used among 26.4% participants with CHD and 24.3% participants with stroke. Any blood pressure lowering medications were used among 65.6% of CHD patients and among 54.8% of stroke patients.

**Table 2:** Use of secondary prevention medications among patients with coronary heart disease (523), stroke (115), and total cardiovascular disease (n = 614).


MEDICATION	CHD (523) N (%) 95% CI	STROKE (115) N (%) 95% CI	TOTAL CVD (614**) N (%) 95% CI

**Antiplatelet drugs**	257 (49.1%) (44.8%–53.5%)	44 (38.3%) (29.4%–47.8%)	292(47.6%) (43.6%–51.6%)

**Statins**	197 (37.7%) (33.5–42%)	39 (33.9%) (25.4%, 43.3%)	228 (37.1%) (33.3%–41.1%)

**Beta blockers**	216 (41.3%) (37.1%–45.7%)	30 (26.1%) (18.3%–35.1%)	239 (38.9%) (35.1%–42.9%)

**ACE inhibitors or ARBs**	138 (26.4%) (22.7%–30.4%)	28 (24.3%) (16.8%–33.2%)	155 (25.2%) (21.9%–28.9%)

**Diuretics**	66 (12.6%) (9.9%–15.8%)	14 (12.2%) (6.8%–19.6%)	74 (12.1%) (9.6%–14.9%)

**Calcium-channel blockers**	97 (18.6%) (15.3%–22.2%)	21 (18.3%) (11.7%–26.6%)	109 (17.8%) (14.8%–21.0%)

**Any Blood Pressure lowering medications** ^*^	343 (65.6%) (61.3%, 69.7%)	63 (54.8%) (45.2%–64.1%)	385 (62.7%) (58.7%–66.5%)


*Any blood pressure lowering medications included using at least Beta blockers, Angiotensin Converting Enzyme-inhibitors (ACE-I), Angiotensin Receptor Blockers (ARBs), diuretics or Calcium channel blockers. **24 patients had both stroke and CHD.

There was no statistically significant difference between the urban and the rural areas in the proportion of medication use for any of the medication classes except for beta-blockers which was 44.7% in urban compared to 13.6% in rural KSA (p = 0.015) (**Supplementary Table 2**).

There were, however, differences in medication use between countries. Antiplatelet use was highest in UAE 47 (65.2%) and KSA 39 (56.5%), followed by Iran 167 (46.5%), and was lowest in the OPT 39 (34.2%). Beta-blocker use was 25 (34.7%) in UAE, 24 (34.8%) in KSA, 24 (21.1%) in OPT and highest 166 (46.2%) in Iran. ACE inhibitors or ARBs use was highest in UAE 29 (40.3%) but only 20 (29%) in KSA, 34 (29.8%) in OPT and 72 (20%) in Iran. Statin use was high in UAE and KSA 41 (56.9%) and 37 (53.6%) respectively, while in OPT it was 45 (39.5%) and in Iran 105 (29.2%). Use of any antihypertensive medications was 51 (70.8%) in UAE, 41 (59%) in KSA, 68 (59.6%) in OPT and 225 (62.6%) in Iran.

**Figure 1:**
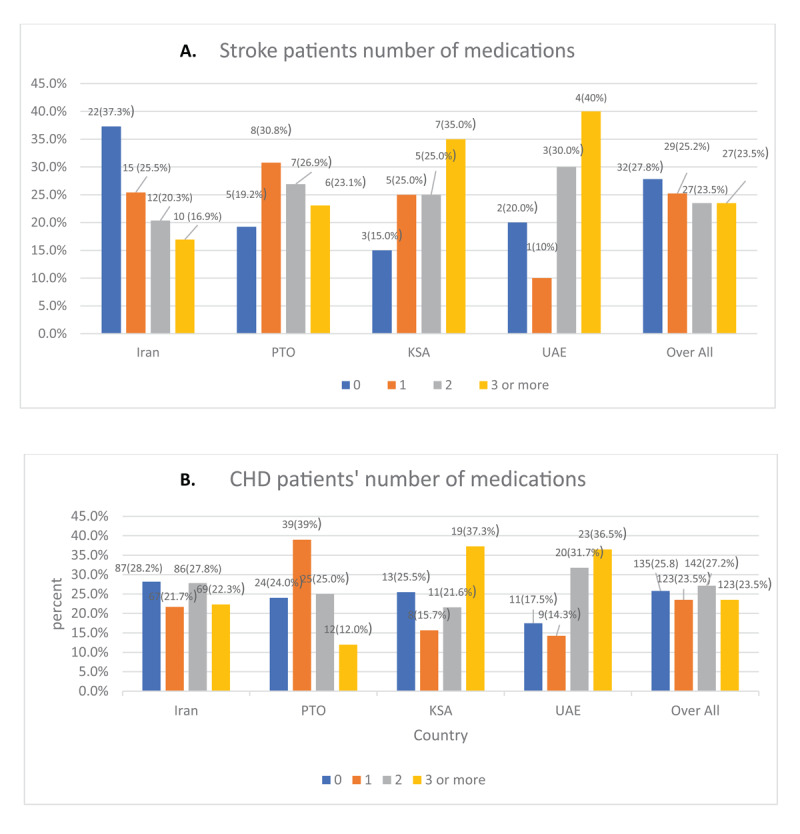
**A.** The number of secondary prevention medications used among participants with stroke, by country and overall. **B.** The number of secondary prevention medications used among participants with CHD, by country and overall. Fisher-Freeman-Halton Exact Test was used to test to compare the distribution of the number of medications between the 4 countries, P- value = 0.425 Chi-squared test was used to compare the distribution of the number of medications between the 4 countries, p-value < 0.001

Figures 1A and 1B present the number of medications used by country. Overall, 27.8% of stroke and 25.8% of CHD patients did not use any medications, and only 23.5% of stroke and 23.5% of CHD patients used three or more medications. The number of medications used by country were also different. Among stroke patients, the use of three or more medications was highest in UAE (40%) and KSA (35%) and lowest in Iran (16.9%), and in the OPT (23.1%). Similarly, among CHD patients, the use of three or more medications was highest in KSA (37.3%) and UAE (36.5%), and lowest in Iran (22.3%) and the OPT (12.0%).

### Independent Risk Factors

Table 3 illustrates adjusted associations (OR, 95%CI) between CVD patient characteristics and medication use in all four countries combined. Increasing age was associated with increasing antiplatelet use (1.05, 1.03–1.08), statin use (1.04, 1.01–1.07), as well as at least one BP lowering medication (1.04, 1.01–1.06), at least one class (1.04, 1.01–1.06) and at least two classes (1.04, 1.02–1.07) of medications.

**Table 3:** Independent factors associated with medication use in individuals with total CVD (CHD and/or stroke).


FACTOR	ANTIPLATELET	BETA-BLOCKER	ACEI/ARB	STATIN	AT LEAST ONE BP LOWERING MEDICATION	AT LEAST ONE CLASS OF MEDICATION	AT LEAST TWO CLASSES OF MEDICATIONS

**Age**	**1.05 (1.03, 1.08)**	0.99 (0.97, 1.01)	1.01 (0.98, 1.04)	**1.04 (1.01, 1.07)**	**1.04 (1.01, 1.06)**	**1.04 (1.01, 1.06)**	**1.04 (1.02, 1.07)**

**Female Gender**	0.8 (0.49, 1.3)	**3.14 (1.89, 5.21)**	0.97 (0.54, 1.74)	1.29 (0.77, 2.17)	**2.73 (1.57, 4.75)**	1.72 (0.98, 3.03)	1.49 (0.91, 2.43)

**Former smoker**	1.24 (0.65, 2.35)	1.6 (0.83, 3.12)	0.78 (0.36, 1.71)	1.1 (0.56, 2.16)	1.3 (0.65, 2.63)	1.14 (0.54, 2.37)	0.99 (0.52, 1.88)

**Current smoker**	1.14 (0.66, 1.98)	1.25 (0.7, 2.22)	1.39 (0.7, 2.74)	1.39 (0.77, 2.51)	1.09 (0.6, 1.96)	0.99 (0.54, 1.81)	1.37 (0.79, 2.39)

**Urban location**	**0.64 (0.42, 0.98)**	1.17 (0.76, 1.81)	1.35 (0.81, 2.24)	0.71 (0.45, 1.11)	0.92 (0.57, 1.47)	0.68 (0.41, 1.1)	0.81 (0.53, 1.24)

**Middle-Household Wealth Index Tertile**	**1.98 (1.17, 3.34)**	1 (0.60, 1.7)	1.15 (0.61, 2.16)	1.26 (0.72, 2.21)	1.6 (0.91, 2.81)	1.41 (0.79, 2.51)	1.37 (0.82, 2.3)

**Highest Household Wealth Index Tertile**	1.8 (0.88, 3.65)	0.89 (0.44, 1.84)	0.78 (0.33, 1.83)	1.34 (0.63, 2.84)	1.08 (0.5, 2.36)	1.51 (0.67, 3.37)	1.12 (0.55, 2.27)

**Secondary higher education**	1.15 (0.68, 1.94)	1.07 (0.62, 1.82)	0.88 (0.46, 1.66)	1.44 (0.82, 2.52)	1.24 (0.7, 2.2)	1.13 (0.63, 2.03)	1.2 (0.7, 2.03)

**College/Trade/University education**	1.34 (0.67, 2.68)	1.29 (0.63, 2.63)	1.23 (0.52, 2.89)	1.85 (0.89, 3.85)	1.82 (0.85, 3.89)	**2.65 (1.15, 6.08)**	1.33 (0.66, 2.7)

**25 <= BMI < 30**	1.09 (0.67, 1.77)	0.87 (0.53, 1.42)	0.58 (0.32, 1.07)	1.44 (0.84, 2.47)	0.77 (0.45, 1.32)	1.16 (0.68, 1.99)	0.88 (0.54, 1.44)

**BMI >= 30**	1.35 (0.8, 2.26)	0.6 (0.35, 1.01)	0.88 (0.48, 1.62)	**1.77 (1.01, 3.1)**	0.96 (0.54, 1.71)	1.26 (0.70, 2.27)	1.12 (0.67, 1.89)

**Hypertension (yes)**	1.12 (0.75, 1.67)	**2.26 (1.49, 3.44)**	**5.75 (3.17, 10.44)**	1.41 (0.92, 2.16)	**5.17 (3.34, 7.99)**	**2.87 (1.83, 4.5)**	**1.88 (1.26, 2.81)**

**Diabetes (yes)**	1.09 (0.74, 1.6)	0.89 (0.60, 1.31)	**1.87 (1.19, 2.94)**	**1.78 (1.2, 2.64)**	1 (0.65, 1.54)	1.51 (0.96, 2.4)	1.23 (0.84, 1.81)

**High-income countries**	**1.66 (1.04, 2.65)**	0.76 (0.47, 1.22)	1.33 (0.8, 2.22)	**2.49 (1.56, 3.98)**	0.85 (0.51, 1.44)	1.15 (0.66, 2.04)	**1.81 (1.13, 2.89)**


Values presented as Odds ratio (95% CI for the Odds ratio). Reference groups are: Male gender, non-smoker, Rural location, lowest. Household Wealth Index Tertile, lowest education, BMI < 25, no hypertension, no diabetes, and lower-income countries. BMI: Body Mass Index. **Bold font** indicates a significant factor (p-value < 0.05).

Female were more likely than males to be on a beta-blocker (3.14,1.89–5.21) and use at least one BP lowering medication (2.73,1.57–4.75). Those with higher education (college/trade/university education) were 2.65 (1.15–6.08) more likely to be on at least one class of CVD secondary prevention medication compared to those with lowest education. Statin use was higher in those who were obese (1.77,1.01–3.1) compared to those with BMI <25. Those patients who were in the middle household wealth index tertile were 1.98 (1.17–3.34) more likely to be using antiplatelet compared to those in the lowest household wealth index. On the other hand, urban dwellers were negatively associated with antiplatelet use compared to the rural location 0.64 (0.42–0.98).

Those with hypertension had increased use of beta-blockers (2.26,1.49–3.44), ACEI/ARB (5.75, 3.17–10.44), at least one BP lowering medication, at least one class (2.87,1.83–4.5), and at least two classes of CVD secondary prevention medications. Diabetic patients with CVD had an increased (1.87,1.19–2.94) ACEI/ARB and statin (1.78,1.2–2.64) use.

Higher-income countries had a higher use of antiplatelet (1.66,1.04–2.65), statin (2.49,1.56–3.98) and at least two classes of CVD secondary prevention medications.

## Discussion

In this prospective cohort of 11,226 participants from four ME countries between 2005 and 2015, 614 (5.5%) had a history of CVD event at baseline (1% stroke, 4.7% CHD and 0.2% had both). After a median of 4 years, one in four participants with CVD (stroke 27.8% stroke and 25.8% CHD) did not use any medications for the secondary prevention of CVD and less than a quarter used three or more medications (23.5% stroke and 23.5% CHD). These proportions are well below the modest target of 50% coverage of drug therapy with three or more drugs set out in the WHO Global Monitoring Framework for Non-Communicable Diseases (25). These rates of use in ME is an overestimate of the total burden, as our analysis includes only those with CVD and excludes all others, including those without CVD but high estimated risk of CVD who are expected to have even lower rates of CVD medication use.

### Comparison with Other Regions

These are better rates of use compared to similar community-based studies. In an earlier study from Golestan in Iran (2004–2008), only 7.3% of patients were on at least three medications and 43.0% did not receive any of the four medications for CVD (26). From the other PURE regional analysis, in South Asia (2003–2009), three or more medications were used in only 2.8% and none in 81.5% (27), while in South America (2003–2009), a substantial proportion of patients did not receive any proven therapy (CHD 31%, stroke 54%) while only a minority of patients received either all 4 (4.1%) or 3 proven therapies (3.3%) (28). In China (2005–2009), nearly 80% of individuals with CHD and 73% of individuals with stroke were not taking any proven secondary prevention medication (29).

### Comparison Within the Four Countries

The number of medications used in our study was greater in UAE and KSA (HIC) compared to OPT and Iran (MIC). It has previously been shown that almost two-thirds of the effect on the variation in the use of these medications is due to the country’s economic status (17).

In the 2018 WHO health system profiles, the out of pocket expenditure is higher in MIC vs HIC (Iran 35% and OPT 45.5%, vs 17.8% UAE and 14.3% in Saudi). Similarly, the Physicians per 10,000 population was lower (Iran 11.4 and OPT 17.7 vs 24.3 and 25.3 in Saudi) (30). Affordability and availability of medications and accessibility of medical facilities within countries of different wealth seems to be one of the important reasons for the differences in CVD secondary prevention medication use (31).

The rates of medication use observed in our study, after a median of four years of CVD events, are much lower compared to the use of these medications at discharge after their event. For instance, after acute coronary syndrome, registry reports from both UAE (22) and Gulf Middle East countries (20,21) showed very high rates of prescription of these medications (for example >90% use of antiplatelet and statin use). This decrease in use at the community level, even in high-income countries like UAE and KSA, needs to be investigated if we want to increase the use of these protective medications in these very high-risk individuals. There are multiple barriers at the patient, clinician, and health system level that contribute to suboptimal CVD secondary prevention (32). This gap has been difficult to close even in other high-income countries (33).

A study from KSA showed that there is generally poor knowledge of secondary prevention guidelines among family physicians (34). Another study from OPT highlighted that conflict has not only impacted on the ability of healthcare providers to deliver care in an environment fraught with barriers, but also the Palestinian people’s access to such healthcare and to be motivated to engage in adopting a healthier lifestyle (35). Accessibility and availability of healthcare facilities and motivation of patients are important factors in adhering to clinical practice guidelines and medication use.

### Stroke versus CHD

A finding in our analysis is the consistent underuse of the medications in stroke compared to CHD. This is not a new finding and has been documented (36). However, this gap is worth noting, and calls for increasing awareness and education among patients and healthcare workers to improve medication use and prevent secondary events not only among CHD but also among stroke patients.

### Independent Predictors of Use

In our analysis, the independent predictors of higher use of CVD secondary prevention medications were increasing age, female gender, higher education, higher wealth in individual households, residence of a higher income country as well as being obese, hypertensive or diabetic. Higher socioeconomic status (household wealth, country wealth and education) and higher contact and accessibility to health care (increasing age, female gender, obesity, diabetes and hypertension) seem to be the most important association themes. Low education in those with CVD (67.1%) as well as in the whole PURE-ME cohort (49.9%) makes it an important negative predictor.

An interesting example of higher contact and accessibility in the study, is the greater usage of antihypertensive medications in females. The awareness rate is higher in women than in men in both high-income countries-HIC (72% women vs 62% men) and middle- and low-income countries-MIC/LIC (45% women vs 31% men), probably due to their earlier exposure to screening during pregnancy and childbirth. Furthermore, in HIC (62% women vs 49% men) and MIC/LIC (36% women vs 22% men), women reported a higher rate of antihypertensive medication use compared with men, and better hypertension control rates HIC (52% women vs 49% men) and MIC/LIC (28% women vs 23% men) (36,37). Similarly, the fact that both hypertensives and diabetics were consistently associated with higher use of medications is attributable to self-awareness of CVD risk and contact with the health system.

### Possible Approaches of Achieving Target

Individual country initiatives like the vision 2030 of Saudi Arabia towards an increasingly educated population and revamped healthcare infrastructure with emphasis on preventative services are important (38). However, achieving the WHO targets is challenging not only for MIC and LIC but also for HIC. The main areas where innovative methods are required are tackling accessibility of health care and making the medications available, affordable and adherable. Two innovative, low cost and simple strategies have shown promise and can rapidly be implemented in most resource settings. Task sharing with non-physician healthcare workers (NPHW) and use of a single pill combination (polypill) therapy have been used successfully in primary and secondary prevention of CVD. For instance, Kaiser Permenante in California used these methods, to substantially increase the hypertension control rates (39). It has also been shown that using NPHW, after a period of training and with the support (treatment algorithms and oversight from physician), can be safe and effective in both primary and secondary CVD prevention (40,41,42,43,44). Similarly, a polypill was shown to reduce CVD events in primary and secondary CVD prevention compared to usual care attributed mainly to increased adherence (45).

Besides national policies towards an educated population and priming the healthcare systems towards prevention, the wide gap of accessibility, affordability and adherence could be bridged by increased use of task sharing with non-physician health workers and the use of polypill. Adapting these methods could help achieve the WHO target even in the low resource countries.

**Limitations:** First, since PURE was a large cohort study, samples were not selected to be nationally representative. However, when compared with national population data, the groups are broadly similar to the general population (46). Second, because the numbers with cardiovascular disease are low in some ME countries we have analysed the predictors from the total CVD from all four participating countries. Third, the diagnosis of CHD and stroke were self-reported as was the use of medication, this comes with its disadvantages. Fourth, our data was collected over a span of 10 years (2005–2015) and rates of use could have changed. However, we have been collecting data not only at baseline but also over several years of follow up (**Appendix I and II**) and intend to publish these trends.

**Strengths:** To our knowledge, this is the largest multinational study in the ME to assess the use of secondary prevention medications in individuals with a history of CHD or stroke. Hospital or general practice registries tend to overestimate the rates of actual use of secondary prevention drugs in a population, our data however, is community based and our methods and questionnaires were all standardized across all nations. This study, therefore, provides a more realistic estimation of medication use. Several regions within PURE study can also be compared as the same standardized methods were used.

## Conclusions

The use of secondary prevention medication in these four ME countries has not reached the modest recommended WHO targets. Independent factors of higher use were, better socioeconomic status (household wealth, country wealth and education) and better contact and accessibility to health care (increasing age, female gender, obesity, diabetes and hypertension).

## Data Accessibility Statement

All data generated or analyzed during this study are included in this published article [and its supplementary information files]. The Population Health Research Institute (PHRI) is the sponsor of this study. The PHRI believes the dissemination of research results is vital and sharing of data is important. PHRI prioritizes access to data to researchers who have worked on the research study for a significant duration, have played substantial roles, and have participated in raising the funds to conduct the study. Data will be disclosed only upon request and approval of the proposed use of the data by a Review Committee. Specific collaborative projects can be developed with groups with similar data for joint analyses. Data are available to the journal for evaluation of reported analyses. Data requests from other investigators will not be considered until 5 years after the date of publication.

## Additional Files

The additional files for this article can be found as follows:

10.5334/gh.1349.s1 Supplementary Tables. HFrEF polypill stakeholder survey with modified implementation science outcome measures.

10.5334/gh.1349.s2 Appendix I. Adult Questionnaire.

10.5334/gh.1349.s3 Appendix II. Individual Contact Form.
