# Supplementary material for: Use of Cardiovascular Disease Secondary Prevention Medications in Four Middle East Countries in a Community Setting
Source: Glob Heart. 2024 Aug 26;19(1):70. doi: 10.5334/gh.1349 (PMC11363892; doi:10.5334/gh.1349)
Supplement: Supplementary Tables. — HFrEF polypill stakeholder survey with modified implementation science outcome measures. [file gh-19-1-1349-s1.pdf]

Supplementary Tables

Country	Overall n	Community Location		Gender		Stroke n (%)	Coronary Heart Disease n (%)	Total CVD n (%)
		Urban n (%)	Rural n (%)	Female n (%)	Male n (%)			
**UAE**	1499	995 66.4%	504 33.6%	982 65.5%	517 34.5%	10 0.7%	63 4.2%	72 4.8%
**KSA**	2046	1545 75.5%	501 24.5%	882 43.1%	5409 56.9%	20 1.0%	51 2.5%	69 3.4%
**OPT**	1668	910 54.6%	758 45.4%	817 49%	851 51%	26 1.6%	100 6.0%	114 6.8%
**Iran**	6013	3031 50.4%	2982 49.6%	3138 52.2%	2875 47.8%	59 1.0%	309 5.1%	359 6.0%
**All**	11226	6481 57.7%	4745 42.3%	5817 51.8%	5409 48.2%	115 1.0%	523 4.7%	614 5.5%

Supplementary Table I: Characteristics of study participants in four Middle East countries overall, by gender, community location and cardiovascular disease. (n=11,226)

**Class of Medication**	**UAE N=72**		**Saudi Arabia N=69**		**OPT N=114**		**Iran N=359**	
	Urban<br>45	Rural<br>27	Urban<br>47	Rural<br>22	Urban<br>54	Rural<br>60	Urban<br>175	Rural<br>184
**Antiplatelet drugs**	27<br>60.0%<br>(44.3%-<br>74.3%)	20<br>74.1%<br>(53.7%-<br>88.9%)	28<br>59.6%<br>(44.3%-<br>73.6%)	11<br>50.0%<br>(28.2%-<br>71.8%)	14<br>25.9%<br>(15%-<br>39.7%)	25<br>41.7%<br>(29.1%-<br>55.1%)	79<br>45.1%<br>(37.6%-<br>52.8%)	88<br>47.8%<br>(40.4%-<br>55.3%)
	47(65.2%)		39(56.5%)		39(34.2%)		167(46.5%)	
**Beta blockers**	16<br>35.6%<br>(21.9%<br>-<br>51.2%)	9<br>33.3%<br>(16.5%-<br>54%)	21<br>44.7%<br>(30.2 -<br>59.9%)	3<br>13.6%<br>(2.9%-<br>34.9%)	11<br>20.4%<br>(10.6%-<br>33.5%)	13<br>21.7%<br>(12.1%-<br>34.2%)	75<br>42.9%<br>(35.4%-<br>50.5%)	91<br>49.5%<br>(42.0%-<br>56.9%)
	25(34.7%)		24(34.8%)		24(21.1%)		166(46.2%)	
**ACE inhibitors or ARBs**	16<br>35.6%<br>(21.9%-<br>51.2%)	13<br>48.1%<br>(28.7%-<br>68.1%)	14<br>29.8%<br>(17.3%-<br>44.9%)	6<br>27.3%<br>(10.7%-<br>50.2%)	20<br>37.0%<br>(24.3%-<br>51.3%)	14<br>23.3%<br>(13.4%-<br>36%)	35<br>20.0%<br>(14.3%-<br>26.7%)	37<br>20.1%<br>(14.6%-<br>26.6%)
	29(40.3%)		20(29%)		34(29.8%)		72(20%)	
**Statins**	27<br>60.0%<br>(44.3%-<br>74.3%)	14<br>51.9%<br>(32%-<br>71.3%)	26<br>55.3%<br>(40.1%-<br>69.8%)	11<br>50.0%<br>(2.9%-<br>34.9%)	17<br>31.5%<br>(19.5%-<br>35.6%)	28<br>46.7%<br>(33.7%-<br>60.0%)	48<br>27.4%<br>(21%-<br>34.7%)	57<br>31.0%<br>(24.4%-<br>38.2%)
	41(56.9%)		37(53.6%)		45(39.5%)		105(29.2%)	
**Blood Pressure lowering drugs**	29<br>(64.4%)<br>(48.8%-<br>78.1%)	22<br>(81.5%)<br>(61.9%-<br>93.7%)	30<br>(63.8%)<br>(48.5%-<br>77.3%)	11<br>(50.5%)<br>(28.2%-<br>71.7%)	35<br>(64.8%)<br>(50.6%-<br>77.3%)	33<br>(55.0%)<br>(41.6%-<br>67.9%)	104<br>(59.4%)<br>(51.8%-<br>66.8%)	121<br>(65.8%)<br>(58.4%-<br>72.6%)
	51(70.8%)		41(59%)		68(59.6%		225(62.7%)	

No statistical differences between rural and urban use of medication except with Beta Blockers usage in KSA (p-value = 0.015)

Supplementary Table 2. Medication usage in individuals with total CVD (614) by country and community location.
