# Supplementary material for: Use of Cardiovascular Disease Secondary Prevention Medications in Four Middle East Countries in a Community Setting
Source: Glob Heart. 2024 Aug 26;19(1):70. doi: 10.5334/gh.1349 (PMC11363892; doi:10.5334/gh.1349)
Supplement: Appendix I. — Adult Questionnaire. [file gh-19-1-1349-s2.pdf]

Subject ID

Centre #	*Community#*	*Household #*	*Subject #*	**Subject Initials** *F M L*

17. Have you ever been diagnosed with any of the following?(check all that apply)

	No	Yes	#of yrs since diagnosis
a) Diabetes	☐	☐	
b) Hypertension/ high blood pressure	☐	☐	
c) Stroke	☐	☐	
d) Angina/heart attack/ Coronary artery disease	☐	☐	
e) Heart failure	☐	☐	
f) Other heart disease	☐	☐	
h) Hepatitis/Jaundice	☐	☐	
g) Cancer	☐	☐	
i) COPD	☐	☐	
j) Asthma	☐	☐	
k) Tuberculosis	☐	☐	
l) Malaria	☐	☐	

Please refer to facing page for cancer sites ☐☐ *site* ____________ *other, specify*

18. Have you been taking any medications regularly (ie. at least once per week) in the last month? ☐ No → **go to 19** ☐ Yes

a) If yes, for what conditions:

	No	Yes
Blood pressure	☐	☐
Cholesterol lowering drugs	☐	☐
Stroke	☐	☐
Diabetes	☐	☐
Asthma	☐	☐
Others	☐	☐ → If Yes, specify ____________________
Unknown	☐	☐

Subject ID

☐☐ Centre # ☐☐☐ Community# ☐☐☐ Household # ☐☐ Subject #

Subject Initials ☐☐☐ F M L

18b) List all the medications you are <u>currently</u> consuming at least once a week for the last month?

i) ____________________ ii) ____________________

iii) ____________________ iv) ____________________

v) ____________________ vi) ____________________

vii) ____________________ viii) ____________________

<u>Men go to question #23</u>

<u>For Women Only (Questions 19 - 22)</u>

19. Are you currently pregnant ? ☐ No ☐ Yes → **Go to #21**

20. Do you still have periods? ☐ No → **(answer 20a)** ☐ Yes → **Go to #21**

a) How many years since you stopped menstruating? ☐☐ years

21. Have you ever used an oral contraceptive? ☐ No ☐ Yes

22a) How many live children have you given birth to? ☐☐ Boys ☐☐ Girls

b) Did you breast feed any of your children? ☐ No ☐ Yes
