# Supplementary material for: Use of Cardiovascular Disease Secondary Prevention Medications in Four Middle East Countries in a Community Setting
Source: Glob Heart. 2024 Aug 26;19(1):70. doi: 10.5334/gh.1349 (PMC11363892; doi:10.5334/gh.1349)
Supplement: Appendix II. — Individual Contact Form. [file gh-19-1-1349-s3.pdf]

Appendix II

PURE — Individual Contact Form — CRF 371

Subject ID: [ ][ ] Centre # [ ][ ][ ] Community # [ ][ ][ ] Household # [ ] Member #

Subject Initials: [ ] F [ ] M [ ] L

Did any of the following **events/new diagnoses** occur since the last follow-up?

	No	Yes		Report No.	Report No.	Report No.
**5a.** MI/Heart Attack	☐	☐ →	**Complete MI Report, CRF 47-49**	☐☐☐	☐☐☐	☐☐☐
**5b.** Stroke	☐	☐ →	**Complete Stroke Report, CRF 42-44**	☐☐☐	☐☐☐	☐☐☐
**5c.** Angina	☐	☐ →	**Complete Angina Report, CRF 117-118**	☐☐☐	☐☐☐	☐☐☐
**5d.** Heart Failure	☐	☐ →	**Complete Heart Failure Report, CRF 30-33**	☐☐☐	☐☐☐	☐☐☐
**5e.** Atrial Fibrillation/ Flutter	☐	☐ →	**Complete AFib Report, CRF 122-124**	☐☐☐	☐☐☐	☐☐☐
**5f.** Cancer	☐	☐ →	**Complete Cancer Report, CRF 40-41**	☐☐☐	☐☐☐	☐☐☐
**5g.** Tuberculosis	☐	☐ →	**Complete TB Report, CRF 57-58**	☐☐☐	☐☐☐	☐☐☐
**5h.** HIV/AIDS	☐	☐ →	**Complete HIV/ AIDS Report, CRF 64 <u>for NEW diagnoses ONLY</u>**	**OR** → ☐ Not Answered		
**5i.** Malaria	☐	☐ →	**Complete Malaria Report, CRF 67**	☐☐☐	☐☐☐	☐☐☐
**5j.** COPD/Chronic Bronchitis/Emphysema	☐	☐ →	**Complete COPD/Chronic Bronchitis/Emphysema Report, CRF 79 <u>for NEW diagnoses ONLY</u>**			
**5k.** Asthma	☐	☐ →	**Complete Asthma Report, CRF 76 <u>for NEW diagnoses ONLY</u>**			
**5l.** Pneumonia	☐	☐ →	**Complete Pneumonia Report, CRF 73**	☐☐☐	☐☐☐	☐☐☐
**5m.** Renal/Kidney (Dialysis/Transplant/Failure)	☐	☐ →	**Complete Kidney Report, CRF 131**	☐☐☐	☐☐☐	☐☐☐

6. Since the <u>last follow-up</u>, have you had any injuries that were serious enough to limit your normal activities for at **least one** day? ☐ No ☐ Yes → **Complete Injury Report, CRF 95-96** — Report No. ☐☐☐ Report No. ☐☐☐ Report No. ☐☐☐

7. Have you been hospitalized for any other reason(s) <u>aside from</u> the events/diagnoses listed in **Q5 and 6** above? ☐ No ☐ Yes → **Complete Hospitalization Report, CRF 82** — Report No. ☐☐☐ Report No. ☐☐☐ Report No. ☐☐☐

			<u>**Date of Diagnosis**</u>
**8.** Have you ever been diagnosed with Valvular Heart Disease?	☐ No	☐ Yes →	☐☐☐☐ *year* ☐☐ *month* ☐ Unk
**9.** Have you ever been diagnosed with Atrial Fibrillation / Atrial Flutter?	☐ No	☐ Yes →	☐☐☐☐ *year* ☐☐ *month* ☐ Unk
**10.** Have you ever been diagnosed with Crohn's Disease?	☐ No	☐ Yes →	**Complete IBD Event Report, CRF 135 <u>for NEW diagnoses ONLY</u>**
**11.** Have you ever been diagnosed with Ulcerative colitis?	☐ No	☐ Yes →	**Complete IBD Event Report, CRF 135 <u>for NEW diagnoses ONLY</u>**

FOR REFERENCE ONLY
USE TABLET TO COLLECT DATA

Subject ID: ☐☐ ☐☐☐ ☐☐☐ ☐
Centre # *Community #* *Household #* *Member #*

Subject Initials: ☐☐☐
F M L

Subject Medical History

12. Have you experienced any of the following in the *last six months*?

	No	Yes
**a)** Chest pain or tightness with usual activity	☐	☐
**(i) If Yes,** does the pain spread to the back, neck or inner border of arm	☐	☐
**b)** Breathlessness with usual activity	☐	☐
**c)** Cough for at least 2 weeks	☐	☐
**d)** Any sputum while coughing	☐	☐
**e)** Blood in sputum	☐	☐
**f)** Wheezing or whistling in the chest	☐	☐
**g)** Early morning cough with chest tightness	☐	☐
**h)** Involuntary weight loss of > 3kg	☐	☐
**i)** Bloody stools	☐	☐
**j)** Abdominal pain at least 3 times a week for at least 4 weeks	☐	☐
**k)** Diarrhea *(more than 3 bowel movements daily)* for 7 consecutive days	☐	☐
**l)** Night-time diarrhea or abdominal pain (wakes you from sleep)	☐	☐

13. Have you had a cough with sputum for 3 months each year, in at least the *last 2 years*? ☐ No ☐ Yes

14. Have you ever fractured a bone? ☐ No → **Go to Q15.** ☐ Yes → **Complete Q14a-c below.**

a) Number of fractures ☐☐

b) Years since last fracture ☐☐ yrs.

c) Bone(s) broken in the most recent fracture:
Note: if more than 3, list most severe sites
Refer to facing page for codes

☐ **If other, specify** → ______________________

☐ → ______________________

☐ → ______________________

FOR REFERENCE ONLY
USE TABLET TO COLLECT DATA

PURE Individual Contact Form CRF 381

Subject ID: ☐☐ ☐☐☐ ☐☐☐ ☐
Centre # Community # Household # Member #

Subject Initials: ☐☐☐
F M L

31. Do you have **hypertension/high blood pressure**? ☐ No → **Go to Q32.** ☐ Yes → **Go to Q31a.**

31a. Are you taking medications regularly to lower your blood pressure? ☐ No
☐ Yes → **If Yes, list in Q36.**

32. Do you have **diabetes**? ☐ No → **Go to Q33.** ☐ Yes → **Go to Q32a.**

32a. Was it diagnosed since last follow-up? ☐ No → **Go to Q32b.** ☐ Yes → **Complete Diabetes Report CRF 87; Proceed to Q32b.**

32b. Are you taking medications regularly for diabetes? ☐ No
☐ Yes → **If Yes, list in Q36.**

33. Do you have high **cholesterol**? ☐ No → **Go to Q34.** ☐ Yes → **Go to Q33a.**

33a. Are you taking medications regularly to lower your cholesterol? ☐ No
☐ Yes → **If Yes, list in Q36.**

34. Are you taking **aspirin** regularly? ☐ No ☐ Yes → **If Yes, list in Q36.**

35. Are you taking **any other heart medications**? ☐ No ☐ Yes → **If Yes, list in Q36.**

36. List **ALL medications** the participant is taking regularly. **OR** → ☐ Participant is **NOT** regularly taking any medications

(Note: This should include ALL medications for conditions listed above plus any other medications the participant is taking regularly, for any reason)

	Code			Code	
i)	☐☐	____	ii)	☐☐	____
iii)	☐☐	____	iv)	☐☐	____
v)	☐☐	____	vi)	☐☐	____
vii)	☐☐	____	viii)	☐☐	____
ix)	☐☐	____	x)	☐☐	____
xi)	☐☐	____	xii)	☐☐	____
xiii)	☐☐	____	xiv)	☐☐	____
xv)	☐☐	____	xvi)	☐☐	____
xvii)	☐☐	____	xviii)	☐☐	____

FOR REFERENCE ONLY
USE TABLET TO COLLECT DATA
